# Epidemiology and screening for renal cancer

**DOI:** 10.1007/s00345-018-2286-7

**Published:** 2018-04-02

**Authors:** Sabrina H. Rossi, Tobias Klatte, Juliet Usher-Smith, Grant D. Stewart

**Affiliations:** 1 Academic Urology Group, University of Cambridge, Addenbrooke’s Hospital, Cambridge Biomedical Campus, Hills Road, Box 43, Cambridge, CB2 0QQ UK; 2 Department of Urology, Addenbrooke’s Hospital, Cambridge University Hospitals NHS Foundation Trust, Cambridge, CB2 0QQ UK; 3 The Primary Care Unit, Department of Public Health and Primary Care, University of Cambridge, Cambridge, CB2 0SR UK

**Keywords:** Renal cell carcinoma, Screening, Ultrasound, Early detection, Review

## Abstract

**Purpose:**

The widespread use of abdominal imaging has affected the epidemiology of renal cell carcinoma (RCC). Despite this, over 25% of individuals with RCC have evidence of metastases at presentation. Screening for RCC has the potential to downstage the disease.

**Methods:**

We performed a literature review on the epidemiology of RCC and evidence base regarding screening. Furthermore, contemporary RCC epidemiology data was obtained for the United Kingdom and trends in age-standardised rates of incidence and mortality were analysed by annual percentage change statistics and joinpoint regression.

**Results:**

The incidence of RCC in the UK increased by 3.1% annually from 1993 through 2014. Urinary dipstick is an inadequate screening tool due to low sensitivity and specificity. It is unlikely that CT would be recommended for population screening due to cost, radiation dose and increased potential for other incidental findings. Screening ultrasound has a sensitivity and specificity of 82–83% and 98–99%, respectively; however, accuracy is dependent on tumour size. No clinically validated urinary nor serum biomarkers have been identified. Major barriers to population screening include the relatively low prevalence of the disease, the potential for false positives and over-diagnosis of slow-growing RCCs. Individual patient risk-stratification based on a combination of risk factors may improve screening efficiency and minimise harms by identifying a group at high risk of RCC.

**Conclusion:**

The incidence of RCC is increasing. The optimal screening modality and target population remain to be elucidated. An analysis of the benefits and harms of screening for patients and society is warranted.

**Electronic supplementary material:**

The online version of this article (10.1007/s00345-018-2286-7) contains supplementary material, which is available to authorized users.

## Introduction

Renal cell carcinoma (RCC) is the 9th most common cancer in men and 14th most common cancer in women worldwide [1]. RCC is the most lethal urological malignancy, yet risk factors for the disease have not been completely elucidated [2, 3]. Screening for RCC remains an attractive prospect; however, the ideal screening modality and screening strategy have yet to be determined. This review summarises the epidemiology of RCC and current evidence base on screening, including potential screening modalities, target populations and risk prediction models to aid early detection.

## Methods

We systematically searched the Medline database up to November 2017 to identify studies on screening for RCC. In addition, a separate search was performed to identify studies reporting risk prediction models for the development of RCC in asymptomatic individuals. The full details of the keywords and subject headings used are available in Table S1 (supporting information). The search was limited to English language and human studies. The reference lists of relevant articles were reviewed manually. Studies were included if they reported risk of RCC in adults representative of the general population. We excluded studies reporting data on symptomatic individuals and those pooling renal and urothelial cancers as the outcome.

Furthermore, to include the most contemporary data on the epidemiology of RCC in the United Kingdom, we obtained RCC incidence and mortality data for 1993–2014 by querying the online database of Cancer Research UK (http://www.cancerresearchuk.org/health-professional/cancer-statistics/statistics-by-cancer-type/kidney-cancer, access: 3 January 2018). Age-standardised incidence and mortality rates were extracted per 100,000 population. Trends in overall RCC incidence and according to age and gender were analysed with joinpoint regression models (Joinpoint 4.1; IMS, Calverton, United States). Up to five joinpoints were allowed for trends. Trends during time periods were described as annual percentage change (APC).

## Results

### Renal cancer epidemiology

The incidence of RCC is increasing worldwide and is positively correlated with gross domestic product per capita [4]. Incidence is highest in developed countries, with rates 15-fold higher in North America, Northern and Eastern Europe compared to Africa and South-East Asia [1]. Established risk factors for RCC include increasing age, smoking, obesity, and hypertension (Table 1) [5–7]. The rising incidence of RCC in rapidly developing countries may be partially attributable to increases in these established risk factors, as well as increased detection of incidental malignancy identified with the widespread use of imaging modalities for other abdominal complaints [1, 8, 9]. The proportion of all RCC diagnosed incidentally is now over 50% [10, 11]. It is estimated that 43% of Medicare beneficiaries aged 65–85 years in the USA undergo either a CT chest or CT abdomen over a 5-year period [12]. This “unsystematic screening” has resulted in a size and stage migration towards smaller RCC, with an associated improvement in survival in many developed countries [10].

**Table 1:** Risk factors for renal cell carcinoma (RCC)

Risk factor	Comment
Established risk factors	
Male gender	Positive association [1, 86]
Age	Positive association [1]
Obesity	Positive association with a dose response [5, 86]
Smoking	Positive association with a dose response [86]
Hypertension	Positive association with a dose response. Effect of hypertensive medication on renal cancer risk remains unclear [86, 87]
Renal disease	Increased risk of renal cancer in acquired cystic kidney disease, end-stage renal disease, renal transplant
Alcohol	Moderate alcohol intake has a protective effect relative to abstinence. There is no additional benefit for higher consumption [88–90]
Family history	Affected first-degree relative confers a risk of renal cancer. A number of inherited rare genetic conditions also predispose to renal cancer, including von Hippel–Lindau, hereditary papillary renal carcinoma, Birt–Hogg–Dubé syndrome, hereditary leiomyomatosis renal cell carcinoma, succinate dehydrogenase renal cell carcinoma, and tuberous sclerosis. [91]
Risk factors that are less well characterised	
Physical activity	High/strenuous physical activity is protective [92]
Diabetes	Positive association [93]
Occupational exposure	Trichloroethylene is considered a carcinogenic agent with sufficient evidence for the development of renal cancer according to the International Agency for Research on Cancer [94, 95]. Arsenic and inorganic arsenic compounds, cadmium and cadmium compounds, perfluorooctanoic acid printing processes and welding fumes have limited evidence according to the International Agency for Research on Cancer [95]
Gamma radiation and X radiation	Carcinogenic agent with sufficient evidence in humans according to the International Agency for Research on Cancer [95]
Analgesic use	Meta-analyses suggest acetaminophen is associated with a significant risk of developing kidney cancer. Conflicting results are available regarding non-aspirin NSAIDs. Aspirin did not demonstrate a significant association [96, 97]

*NSAIDs* non-steroidal anti-inflammatory drugs

Mortality rates are stable or decreasing in the majority of Western countries, however, the decline is more pronounced in Western compared to Eastern Europe and North compared to South America [4]. RCC mortality continues to rise in Eastern Europe, however [4]. Renal cancer contributes to a greater average number of years of life lost (a measure of cancer burden dependent on patient age at death and the number of deaths at each age) than both colorectal and prostate cancer [13, 14].

**Fig. 1:**
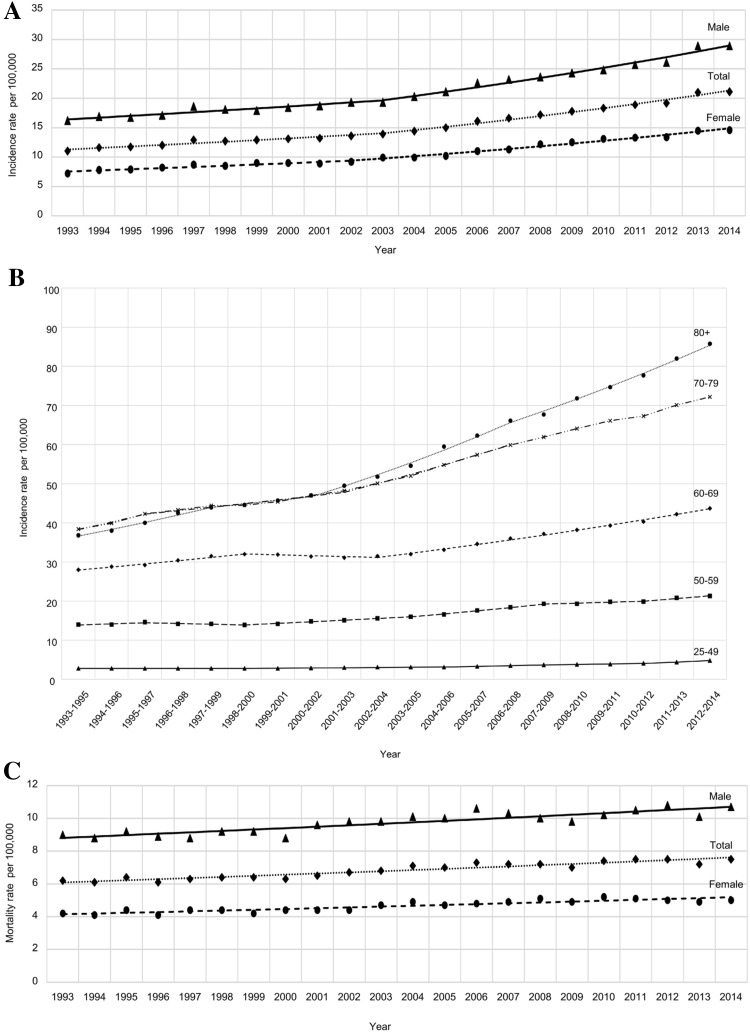
Age-standardised renal cell carcinoma incidence rates according to gender (**a**) and age group (**b**) in the UK population between 1993 and 2014. Incidence rates rose continuously (average annual percentage change 3.1%), especially in the elderly. In contrast, mortality rates (**c**) increased only to a minor extent (average annual percentage change 1.1%), indicating improvements in relative survival

UK figures on RCC incidence and mortality are shown in Fig. 1. Overall, the age-standardised RCC incidence rate increased by 3.1% per year (95% CI 2.8–3.4%) from 1993 through 2014. The overall APC was 2.2% between 1993 and 2003 and 3.9% between 2003 and 2014. Both males and females demonstrated a comparable increase (Fig. 1a). The increase in RCC incidence rates was greatest in older age groups (Fig. 1b). In fact, the average APC was 2.9% (95% CI 2.2–3.5%) in individuals aged 25–49 years, 3.4% for individuals aged 70–79 year and 4.6% in patients aged > 80 years. In contrast to incidence, mortality rates increased only to a minor extent (average annual percentage change 1.1% [95% CI 0.9–1.2%], Fig. 1c), suggesting improvements in relative survival.

### Rationale for screening

Early diagnosis and screening for RCC has been identified as a key research priority within this disease [15]. Despite this, relatively little research has been published regarding screening for RCC over the last decade. RCC fulfils many of the Wilson and Jungner criteria for suitability for screening, however, a number of key uncertainties require further research (Table 2) [14]. Overall survival from RCC is poor, with a 47% 5-year age-standardized relative survival rate in the United Kingdom. Over a quarter of individuals diagnosed with RCC have evidence of metastases at presentation and 5-year age-standardized relative survival rate for stage IV disease is 6% compared to 84% in stage I [16]. Incidentally detected tumours are generally smaller in size and are associated with improved survival relative to symptomatic tumours, independent of tumour grade and stage [17, 18]. A screening programme may improve survival outcomes through earlier detection and treatment of RCC at a curable stage. RCC is generally considered a “surgical disease”; management is operative in all but the most advanced cases, where systemic therapies may prolong life but not provide a cure [19, 20]. As such, early diagnosis is paramount to optimizing survival [19]. Early detection of smaller tumours may allow increasing use of minimally invasive techniques such as robotic or laparoscopic partial nephrectomy and tumour ablation, reducing rates of open surgery with associated high morbidity and hospital stay [21–24]. Modern systemic therapies used in the treatment of metastatic RCC, such as sunitinib, pazopanib, axitinib and nivolumab, are highly expensive and the median cost of anticancer drugs is rising, as is patient life expectancy, and therefore, duration of treatment [25, 26]. It has been postulated that screening for RCC may be a cost-effective strategy through downstaging the disease, reducing the prevalence of metastatic tumours and associated expenditure relating to systemic therapies. However, the ideal screening modality is yet to be determined.

**Table 2:** Wilson and Jungner criteria applied to screening for renal cell carcinoma (RCC) [14]

Criteria for screening	Application to RCC screening
The condition sought should be an important health problem	Renal cancer is the 7th most common cancer in Europe [98]
There should be an accepted treatment for patients with recognised disease	Detection of smaller tumours may preferentially allow minimally invasive techniques reducing rates of open surgery, and therefore, associated morbidity and length of hospital stay
Facilities for diagnosis and treatment should be available	In a health service with a finite budget, important considerations must be made regarding the cost of investigations and management of patients found to have benign SRMs on screening
There should be a recognisable latent or early symptomatic stage	The sojourn time of RCC is between 3.7 and 5.8 years, suggesting that most RCCs have a detectable preclinical period [44]
There should be a suitable test of examination	Focused renal ultrasound thus far represents the only validated screening tool, with high sensitivity (82–83.3%) and specificity (98–99.3%) [56, 57]. Accurate and inexpensive, non-invasive methods of renal cancer detection, using blood or urine as the substrate, remain a research priority
The test should be acceptable to the population	Ultrasound is non-invasive and well tolerated by the general population. AAA screening is performed with ultrasound and attendance rates are 84–85%, with similar rates expected for RCC. [48, 49]
The natural history of the condition, including development from latent to declared disease, should be adequately understood	Reliable clinical predictors of a tumour’s growth rate and aggressiveness are not available Advances have been made in understanding the natural history of small renal masses and the European Active SurveillancE of Renal cancer (EASE study) aims to clarify this further [99]
There should be an agreed policy on whom to treat as patients.	Clear European Association of Urology guidelines have been published regarding the management of RCC [7]
The cost of case finding (including diagnosis and treatment of patients diagnosed) should be economically balanced in relation to possible expenditure on medical care as a whole	A cost-effectiveness analysis is warranted and constitutes a key research priority highlighted in this analysis
Case finding should be a continuing process and not a “once and for all” project	A cost-effectiveness analysis may elucidate the optimal screening frequency, be it one off screening such as AAA, or recurrent screening

*AAA* abdominal aortic aneurysm, *RCC* renal cell carcinoma, *SRM* small renal mass

### Urine dipstick as a screening modality

The incidence of visible and non-visible haematuria is 35% in patients with known RCC, compared to 94% in patients with urothelial carcinoma of the bladder or ureter [27]. Kang et al. reported results of urinary dipstick performed in a screening paradigm in 56,632 asymptomatic healthy individuals aged ≥ 20 years undergoing a “health check-up.” The prevalence of non-visible haematuria at initial urinalysis was 6.2% (3517/56,632), however, in this young, and therefore, low-risk population, only three RCC (prevalence 0.005%) and three bladder cancers were subsequently detected [28]. A feasibility study of population screening utilising home urinary dipstick followed by urinary biomarkers testing in men aged 50–75 years has also been performed. 1747 men were screened but although 23% tested positive for non-visible haematuria, requiring biomarker testing and subsequent imaging, only four bladder and one renal malignancies were detected. One bladder cancer and one renal cancer were missed [29]. Microscopic haematuria is a relatively common and very non-specific finding; therefore, a substantial proportion of individuals screened by dipstick will require further investigation, to detect only a very small number of RCCs. Several other studies have been performed evaluating urine dipstick in screening for renal and bladder cancer, however, the low diagnostic yield and high number of false positives and false negatives preclude this as a screening tool for RCC [29–31].

### Biomarkers as a screening modality

Several serum and urine biomarkers have been proposed as potential screening tools. Soluble urinary proteins are an attractive candidate due to their relative stability and straightforward method of detection via antibody or ligand-based techniques [32]. Perhaps the most promising urinary biomarkers are aquaporin 1 (AQP1) and perilipin 2 (PLIN2). These biomarkers can differentiate RCC from healthy controls, benign renal masses and non-renal urological cancers [33, 34]. Recently, Morrissey et al. evaluated AQP1 and PLIN2 levels prospectively in a screening paradigm in 720 asymptomatic individuals undergoing abdominal CT for a medical reason not related to RCC, 18 patients with histologically proven RCC and 80 self-selected healthy controls. The sensitivity of both biomarkers was 85–92% and the specificity 87–100%; with an area under the ROC of 0.95 and 0.91 for AQP1 and PLIN2, respectively. External validation of these urinary biomarkers in a larger prospective cohort is paramount. However, AQP1 and PLIN2 are markers of clear cell or papillary, but not chromophobe RCC, raising the potential for false-negative results in a screening population. AQP1 levels also correlated with tumour size but not grade, raising the issue of potential detection of indolent renal masses that would never become clinically significant [35, 36]. In addition, evaluation of PLIN2 using Western Blot limits applicability as a screening tool as this is a time consuming, expensive and technically demanding method [37].

Other plasma and urinary biomarkers have also been evaluated. A composite three marker assay [based on nicotinamide *N*-methyltransferase (NNMT), L-plastin (LCP1) and non-metastatic cells 1 protein (NM23A)] was developed and had 90% sensitivity, 95.7% specificity and diagnostic AUC 0.932 for RCC versus healthy controls. However, the assay has limited ability to distinguish RCC from benign renal tumours [38]. Han et al. showed urinary KIM1 is also significantly higher in patients with RCC than controls, however, its use as a diagnostic marker is limited by low specificity [39, 40]. Frantzi et al. demonstrated that though a single urinary peptide with diagnostic value was not identified, a model based on 87 peptides has reported 80% sensitivity and 87% specificity [41]. Accurate and inexpensive, non-invasive methods of renal cancer detection, using blood or urine as the substrate, remain a research priority. Evaluation of cell-free DNA is one such avenue currently under evaluation [42].

### Computed tomography as a screening modality

Although non-contrast CT has not been proposed as a dedicated screening tool for RCC, the value of screening abdominal CT for the simultaneous detection of aortic aneurysms and a variety of solid abdominal organ malignancies has been investigated [43]. Ishikawa et al. screened 4543 healthy individuals aged ≥ 40 years, however, the prevalence of solid organ malignancy was only 0.1% and thus they concluded that screening low-risk individuals was unlikely to be cost-effective [43]. Fenton et al. estimated the pooled prevalence of renal cancer detected in middle-aged American individuals undergoing a variety of screening CT modalities (including whole body CT, CT screening for lung cancer, colorectal cancer and coronary artery disease) as 0.21%, which is substantially higher [44]. Renal lesions are the most common extracolonic finding noted on CT colonography performed during screening for colorectal cancer, suggesting CT colonography may enable early detection of incidental RCC [45]. However, it is widely recognised that CT colonography leads to considerable over-diagnosis of a variety of indeterminate visceral lesions. Extracolonic findings are noted in 40–70% of screening CT colonography. Of these, 5–35% require further imaging or follow-up, but only 3% require treatment, with significant burden on patients and resources [46]. A health economic analysis has demonstrated that whole body CT is not a cost-effective screening intervention due to the high financial burden associated with follow-up for false-positive- and incidental findings [47]. In view of this, it is unlikely non-contrast abdominal CT would ever be recommended for population screening for RCC [46].

### Ultrasound as a screening modality

Ultrasound has arisen as a potential screening tool for RCC as it is a widely utilised, established, inexpensive, non-invasive technique of identifying renal lesions without exposure to radiation. National abdominal aortic aneurysm (AAA) screening programmes in men over the age of 65 years are established in the United Kingdom and Sweden and have demonstrated that an ultrasound-based screening programme can be delivered by trained technicians in a primary care setting [48, 49]. These screening programmes are ideal vehicles to explore the possibility of screening for RCC due to the similarities in risk factors and mode of detection between RCC and AAA [50].

Ultrasound is less sensitive and specific compared to CT for the detection of RCC, with ultrasound detection rates dependent on renal lesion size. Ultrasound enables the detection of 85–100% tumours > 3 cm in size, but only 67–82% of tumours 2–3 cm in size [51–53]. Therefore, ultrasound screening for RCC has the potential to lead to false-negative results in masses < 3 cm in size. Complete diagnostic visualisation of kidneys by ultrasound occurs in 97.4% of cases, comparing favourably with 98.8% visualisation rates of the aorta in AAA screening [54, 55]. Mizuma et al. and Filipas et al. report an excellent sensitivity (82–83.3%) and specificity (98–99.3%) of ultrasound for detecting RCC in the general population as part of a screening intervention [56, 57]. The potential for false-negative results was not based on CT which is generally considered gold standard, but rather repeat ultrasound at a 1-year interval and follow-up via a registry and health records. This may artificially inflate the reported accuracy of ultrasound.

Several observational studies have been published on screening for RCC using ultrasound, however, none have been randomized in design, and all were published more than a decade ago [50, 56–61] (Table 3). Mihara et al. screened 219,640 asymptomatic Japanese individuals selected from the general population (age range 29–70 years) over a 13-year period [60]. RCC was detected in 192 cases: 37.8% of detected tumours were < 25 mm in size and only 19.2% of tumours were > 51 mm. No patients had lymph node or distant metastases. Screen-detected RCC was associated with excellent survival outcomes, with 97.4% cumulative survival rates at 5 years and 94.6% at 10 years. Tosaka et al. retrospectively report the results of 41,364 abdominal ultrasounds performed at their institution, including 20,897 asymptomatic individuals undergoing a routine “health check-up” and 20,467 patients undergoing investigations for a non-urological complaint [62]. 5-year survival in this asymptomatic group of individuals diagnosed with RCC was significantly better than that observed in symptomatic patients diagnosed with RCC at the same institution (94.7 vs 60.9%, *p* < 0.01). Filipas et al. and Malaeb et al. performed focused renal ultrasound screening of the general population in a prospective manner. In the former, screening was performed in 9959 asymptomatic individuals > 40 years recruited from the general population [57]. Eleven individuals were diagnosed with RCC. There was no significant difference in mean tumour size between screen-detected cancers and RCCs diagnosed in a hospital in the same region (incidental and symptomatic RCC detection), however, the authors postulate this may be secondary to the limited sample size and survival data were not reported. Malaeb et al. screened for RCC in asymptomatic veterans in conjunction with established AAA screening [50]. 80% survival was reported in patients with screen-detected RCC at 55-month follow-up. All the individuals who died were stage T3 at diagnosis. Taken together, these studies suggest there may be a potential survival benefit associated with early detection through screening for RCC. However, further evidence is required, utilising robust methodology (such as a randomised control trial with long-term follow-up data in a contemporary, well-defined population) to determine whether screening for RCC is associated with improved survival or whether there is simply a lead time bias.

**Table 3:** Characteristics of studies identifying renal cell carcinoma (RCC) using ultrasound in asymptomatic individuals in a screening paradigm

Study (year)	Country	Data collection dates	Study design	Sample demographics: mean or median age (range), % male	Sample size	Histology proven RCC (prevalence)	% RCCs ≤ 5 cm in size	% RCC with metastases at diagnosis	Outcomes in patients with screen-detected RCC
Fujii (1995) [59]	Japan	April 1985–March 1991	Asymptomatic individuals, employee health check-up	Median 53 years (21–85), 72% male	17,941	20 (0.11%)	NR	NR	NR
Spouge (1996) [58]	Canada	6-month period, not specified	Asymptomatic individuals, employee health check-up for business executives	Mean 46.2 years (29–63), 91% male	1000	4 (0.40%)	NR	0%	PAS: 100% Disease-free survival at 5 years: 100%
Spouge (1996) [58] 2nd sample	Canada	2.5-year period, not specified	Asymptomatic individuals, employee health check-up for business executives	Not reported	7925	23 (0.29%)	NR	NR	NR
Mihara (1999) [60]	Japan	August 1983–March 1996	Asymptomatic screening of general population	Age range 29–70 years, gender not reported	219,640	189 (0.09%)	80.8%	0%	PAS: 98.4% Survival at 5 and 10 years: 97.4 and 94.6%
Tsuboi (2000) [61]	Japan	January 1993–June 1997	Asymptomatic individuals, health check-up for the general population	Age range 15–96, 67% male	60,604	13 (0.02%)	69.2% < 5 cm	NR	PAS: 92.9% Survival NR
Mizuma (2002) [56]	Japan	February 1990–December 1995	Asymptomatic individuals, health check-up for the general population	Mean 47 years (25–84 years), 58% male	16,024	6 (0.04%)	83.3% < 5 cm	16.7%	PAS: 100% Survival at 50 months: 100%
Filipas (2003) [57]	Germany	December 1996 for 13 months and January 1998 for 13 months	Asymptomatic screening of general population, individuals aged > 40 years	Mean 61 years (40–94 years), 49% male	9959	11 (0.11%)	36.4% < 5 cm	18.2%	PAS: 81.8% Survival NR
Malaeb (2004) [50]	USA	1993–1997	Asymptomatic screening of veterans (in conjunction with AAA screen)	Mean 66.2 years (50–79 years), 97% male	6678	15 (0.22%)	46.7%	6.67%	PAS: 68.2% Survival at 55 months: 80%
Tosaka (1990) [62]	Japan	1982–1988	Mixed: asymptomatic individuals (part of health check-up; *n* = 20,897) and patients undergoing abdominal ultrasound for non-urological complaint (*n* = 20,467).	Not reported	41,364	19 (0.05%)	NR	0%	Survival at 5 years following nephrectomy: 94.7%
Haliloglu (2010) [100]	Turkey	March 1995–February 2008	Mixed: asymptomatic individuals (part of health check-up) and patients having ultrasound for LUTS	55 years (33–90 years), 64% male	18,203	36 (0.02%)	83.3%	2.8%	PAS: 48.6% Survival:97.2%

*AAA* abdominal aortic aneurysm, *LUTS* lower urinary tract symptoms; NR not reported, *PAS* proportion of patients with suspected renal cell carcinoma who underwent surgery (comprises partial and radical nephrectomy with curative and non-curative intent), *RCC* renal cell carcinoma, *USA* United States of America

### Optimal screening population

One of the perceived barriers to population screening for RCC is the relatively low prevalence of the disease, with subsequent high cost to society to benefit only a small proportion of individuals. A recent meta-analysis, pooling data from 11 studies on the prevalence of RCC detected by screening ultrasound, estimated that screening 1000 asymptomatic individuals from the general population using ultrasound would allow the detection of between one and two cases of RCC [63]. Several high-risk groups exist, however.

Over 70% of patients with Von Hippel Lindau disease will develop RCC, often at an early age, and these individuals are also at high risk of adrenal and pancreatic tumours [7]. As such, annual surveillance with abdominal ultrasound and MRI is recommended to ensure early detection [64]. Patients with end-stage renal failure (ESRF) have an increased risk of RCC; 5–35 times higher than the general population [65, 66]. This is secondary to the development of acquired cystic kidney disease (ACKD) and the risk is proportional to time on dialysis. There is insufficient evidence regarding whether screening for RCC in patients with ESRF is associated with a survival benefit, due to the significantly reduced baseline life expectancy of this patient group [66–68]. Renal transplant recipients are also at increased risk of RCC both in the native kidneys and in the graft; with rates of RCC 10–100 times higher than the general population [69]. Due to structural differences within the kidneys of patients with ESRF and in renal transplant recipients, the sensitivity and specificity of ultrasound in these individuals remain uncertain, a major determinant of cost-effectiveness [65]. Contrast-enhanced CT, especially in the corticomedullary phase, and non-contrast MRI have higher sensitivity and specificity than ultrasound in detecting and characterizing cystic lesions [70, 71]. European Association of Urology guidelines published in 2005 and updated in 2009 recommended annual ultrasound screening of native kidneys and the graft in allograft recipients [69]. However, a subsequent Markov model simulating annual and biennial screening suggested this is not a cost-effective strategy [65]. The Kidney Disease Improving Global Outcomes (KDIGO) and the American Society of Transplantation found insufficient evidence to recommend screening in renal transplant recipients [72], while the Kidney Health Australia guideline recommends screening only in renal transplant recipients at high risk (past/family history of RCC or analgesic nephropathy; ungraded evidence) [73]. More research is necessary to clarify this.

It has been postulated that established risk factors for RCC may be used to identify individuals in the general population who are at higher risk of the disease. Targeted screening of high-risk individuals may prove to be a cost-effective strategy by maximising benefits and reducing harms of screening [5, 50, 74]. For example, Starke et al. reported data on a group of 925 high-risk asymptomatic individuals identified as high risk based on age (≥ 50 years), smoking (≥ 10 pack-year smoking history) and occupational carcinogen exposure(≥ 15 years exposure). At 6.5-years follow-up, ten patients were diagnosed with RCC, giving a prevalence of 1.1% which is nearly ten times higher than in unselected groups representing the general population [75]. A national population registry including 12.2 million individuals also demonstrated an individual standardized incidence ratio of 2.61 for RCC when a sibling is affected. Despite this familial clustering, there is insufficient evidence to recommend routine screening of individuals with one sibling affected with RCC [76]. Risk prediction models, incorporating family history alongside other risk factors, may allow identification of a high-risk group of individuals who may benefit from screening.

We, therefore, performed a systematic review to identify existing risk prediction models for the development of RCC. A similar approach has been adopted in other disease areas, including melanoma and colorectal cancer [77, 78]. We reviewed 2973 article titles/abstracts. Our findings suggest there are no risk prediction models specific for the development of RCC at present. The only model identified was “Your Disease Risk” (https://siteman.wustl.edu/prevention/ydr/), which predicts the risk of 12 common cancers and six chronic diseases in the USA. However, this tool was created through expert consensus rather than patient-level data and its predictive ability and validity for renal cancer has not been established. The development of validated risk prediction models for RCC is, therefore, needed to explore the potential benefits of targeted screening; however, usefulness may be limited by the absence of risk factors specific to RCC, limiting specificity of the model. In future, it may be feasible to incorporate genomic as well as phenotypic factors in risk prediction models, to increase model accuracy.

### Screening considerations: false positives and over-diagnosis

Potential false negatives, false positives and over-diagnosis have been cited as barriers towards screening. The emotional and psychological patient benefits and harms of RCC screening have yet to be quantified [63]. An evaluation of a screening programme for RCC must take into consideration the impact of incidentally detected benign renal lesions on patients and health services. At present, 15–30% of small renal masses (SRM) are found to be benign following surgical excision [79–81]. Advances in the determination of the aetiology of SRMs, with increased utilization and better interpretation of renal biopsy, may reduce these rates in future [82], as may novel urinary or serum biomarkers. Contrast-enhanced ultrasound (CEUS), an emerging imaging modality, involves the injection of a microbubble contrast agent in addition to conventional ultrasound. Due to its invasive nature and requirements for trained staff it does not represent a candidate for screening. However, a meta-analysis demonstrated a sensitivity of 88% and specificity of 80% in the differential diagnosis of benign and malignant tumours, suggesting there may be a role for CEUS in complementing contrast abdominal CT for differentiation of benign and malignant renal masses in future [83].

Screening for RCC also raises the potential issue of over-diagnosis of slow-growing SRMs that would never become clinically significant [37]. Up to one-third of SRMs exhibit aggressive potential (rapid growth or doubling time < 12 months), with the remainder growing slowly or remaining stable in size [84, 85]. Fenton et al. calculated that the sojourn time (mean duration of the detectable preclinical period) of RCC is between 3.7 and 5.8 years, suggesting that most RCs detected by CT screening among middle-aged Americans are likely to progress to clinical diagnosis [44]. Following advances in our understanding of the natural history of SRMs, active surveillance with delayed intervention, either operative or ablative, has become a solution to reduce over-treatment.

## Conclusion

RCC has a poor prognosis and incidence rates are rising, especially in the elderly population. Although screening for RCC remains an attractive prospect, the optimal screening modality and target population is yet to be determined. The development and validation of risk prediction models for RCC, containing phenotypic and genotypic data, is therefore, needed to explore the potential benefits of targeted screening. Urinary biomarkers constitute a promising future option as an inexpensive, readily accessible screening tool.

More research is required to assess whether screening translates to a survival benefit in the context of such a high number of incidentally detected lesions through the widespread use of abdominal imaging. With ever-increasing demands on health services and a finite budget, it is paramount that a screening intervention is not only effective, but also cost effective. In the absence of randomized control trials, a value of information analysis conducted as part of a cost-effectiveness analysis based on existing data may highlight areas to focus future research efforts. Most importantly, there is an ever-increasing focus on reducing harms associated with screening, and studies are required to quantify the emotional impact of RCC screening on patients, including anxiety and quality of life [12].

## Electronic supplementary material

Below is the link to the electronic supplementary material.

Supplementary material 1 (DOCX 12 kb)
