# Supplementary material for: Epidemiology and screening for renal cancer
Source: World J Urol. 2018 Apr 2;36(9):1341–53. doi: 10.1007/s00345-018-2286-7 (PMC6105141; doi:10.1007/s00345-018-2286-7)
Supplement: Supplementary file 1 — Supplementary material 1 (DOCX 12 kb) [file 345_2018_2286_MOESM1_ESM.docx]

**Supplemental Table S1:**

Table S1 illustrates the review search strategy. Medline was systematically searched, using the Ovid platform. Key words and Medical Subject Headings (MeSH) used are shown, where $ indicates right-hand truncation (i.e., search for variations on a word that are formed with different suffixes) and ? is used to retrieve words with both British and American spelling variations. All searches were limited to humans and publications in the English language.

| Search | Search terms |
| --- | --- |
| Search #1 | Kidney neoplasms (Medical Subject Heading) OR renal cell carcinoma OR RCC OR renal mass$ OR renal cancer OR renal neoplas$ OR renal tumo?r OR renal carcinoma OR kidney cancer OR kidney neoplas$ OR kidney tumo?r OR kidney carcinoma OR hypernephroma OR Grawitz tumor OR renal adenocarcinoma OR oncocytoma  AND  Mass screening (Medical Subject Heading) or screening |
| Search #2 | Kidney neoplasms (Medical Subject Heading) OR renal cell carcinoma OR RCC OR renal mass$ OR renal cancer OR renal neoplas$ OR renal tumo?r OR renal carcinoma OR kidney cancer OR kidney neoplas$ OR kidney tumo?r OR kidney carcinoma OR hypernephroma OR Grawitz tumor OR renal adenocarcinoma OR oncocytoma  AND  Incidental finding (Medical Subject Heading) OR incidental$ OR prevalence ((Medical Subject Heading) OR prevalence |
| Search  #3 | Kidney neoplasms (Medical Subject Heading ) OR renal cell carcinoma OR renal cancer OR carcinoma, renal cell/ OR kidney cancer  AND  Risk factors (Medical Subject Heading) OR risk (Medical Subject Heading ) OR risk assessment (Medical Subject Heading) OR probability (Medical Subject Heading ) OR risk$ OR chance$ OR likelihood$ OR probability  AND  Early detection of cancer (Medical Subject Heading ) OR statistical models (Medical Subject Heading) OR predict$ OR model$ OR score$ |
